# User Experiences of Social Support From Companion Chatbots in Everyday Contexts: Thematic Analysis

**DOI:** 10.2196/16235

**Published:** 2020-03-06

**Authors:** Vivian Ta, Caroline Griffith, Carolynn Boatfield, Xinyu Wang, Maria Civitello, Haley Bader, Esther DeCero, Alexia Loggarakis

**Affiliations:** 1 Lake Forest College Lake Forest, IL United States

**Keywords:** artificial intelligence, social support, artificial agents, chatbots, interpersonal relations

## Abstract

**Background:**

Previous research suggests that artificial agents may be a promising source of social support for humans. However, the bulk of this research has been conducted in the context of social support interventions that specifically address stressful situations or health improvements. Little research has examined social support received from artificial agents in everyday contexts.

**Objective:**

Considering that social support manifests in not only crises but also everyday situations and that everyday social support forms the basis of support received during more stressful events, we aimed to investigate the types of everyday social support that can be received from artificial agents.

**Methods:**

In Study 1, we examined publicly available user reviews (N=1854) of Replika, a popular companion chatbot. In Study 2, a sample (n=66) of Replika users provided detailed open-ended responses regarding their experiences of using Replika. We conducted thematic analysis on both datasets to gain insight into the kind of everyday social support that users receive through interactions with Replika.

**Results:**

Replika provides some level of companionship that can help curtail loneliness, provide a “safe space” in which users can discuss any topic without the fear of judgment or retaliation, increase positive affect through uplifting and nurturing messages, and provide helpful information/advice when normal sources of informational support are not available.

**Conclusions:**

Artificial agents may be a promising source of everyday social support, particularly companionship, emotional, informational, and appraisal support, but not as tangible support. Future studies are needed to determine who might benefit from these types of everyday social support the most and why. These results could potentially be used to help address global health issues or other crises early on in everyday situations before they potentially manifest into larger issues.

## Introduction

Previous research suggests that artificial agents may be a promising source of social support for humans and thus benefit health and well-being. For example, artificial agents may help people cope with loneliness and depressive anxiety that often accompanies severe illness and end-of-life experiences [1,2], improve mood and reduce depression and anxiety symptoms for individuals with dementia [3-5], and increase medication adherence and rehabilitation exercise frequency for individuals with chronic obstructive pulmonary disease by providing reminders and helpful information [6]. In addition, conversational agents have been shown to address social isolation and loneliness in older adults by providing empathic feedback, exercise promotion, and anecdotal stories [7], and Web-based cognitive behavioral therapy (CBT) conversational agents have shown to reduce symptoms of depression and anxiety [5]. However, the bulk of this research has been conducted in the context of social support interventions that specifically address very stressful life events or improving health. Little research has examined everyday social support received from artificial agents, that is, social support as an everyday social interaction rather than a response to very stressful life events or health-related situations [8].

Social support is a complex construct, as it has been defined in many ways [9,10], has been categorized into different forms (eg, behaviors, perceptions) [11] and types (eg, instrumental, appraisal, emotional support) [12], and can come from a variety of sources (eg, friends, family, coworkers). In this paper, we define social support as a social psychological concept that “addresses the mechanisms and processes through which interpersonal relationships protect and help people in their day-to-day lives” [13]. Cutrona and Suhr [14] provide a framework to distinguish between several types of social support: (1) informational support, which refers to providing information or advice; (2) emotional support, which refers to providing expressions that include care, love, empathy, and sympathy; (3) appraisal support, which refers to evaluative feedback regarding skills, abilities, and intrinsic value; (4) companionship support, which refers to the enhancement of one’s sense of belonging; and (5) tangible support, which refers to providing needed goods and services. Despite the various definitions and forms of social support, numerous studies have demonstrated its importance in mental and physical health, as it is an important buffering factor for critical life events, illnesses, trauma, and stress [9,15] and affects one’s well-being in everyday circumstances [16,17].

Social support manifests not only in crises such as health-related or very stressful life events but also in everyday situations and contexts [8], and everyday social support forms the basis for the support received during more stressful situations [18]. Given that social support plays a critical role in health and well-being [9,15-17], it is important to examine the kinds of everyday social support that can be provided by artificial agents. This kind of investigation could allow us to potentially address global health issues or other crises early on in everyday situations before they manifest into larger issues.

As a first step in addressing this gap in the literature, we analyzed the user experiences of a popular companion chatbot (Replika) across two exploratory studies to identify the types of everyday social support that users received based on Cutrona and Suhr’s [14] framework of social support. In Study 1, we analyzed a large dataset of publicly available Replika user reviews. In Study 2, we recruited a sample of Replika users to provide in-depth descriptions of their experience of using Replika. We conducted thematic analysis on both datasets to gain rich and detailed insight of everyday social support received from interactions with Replika.

**Figure 1:**
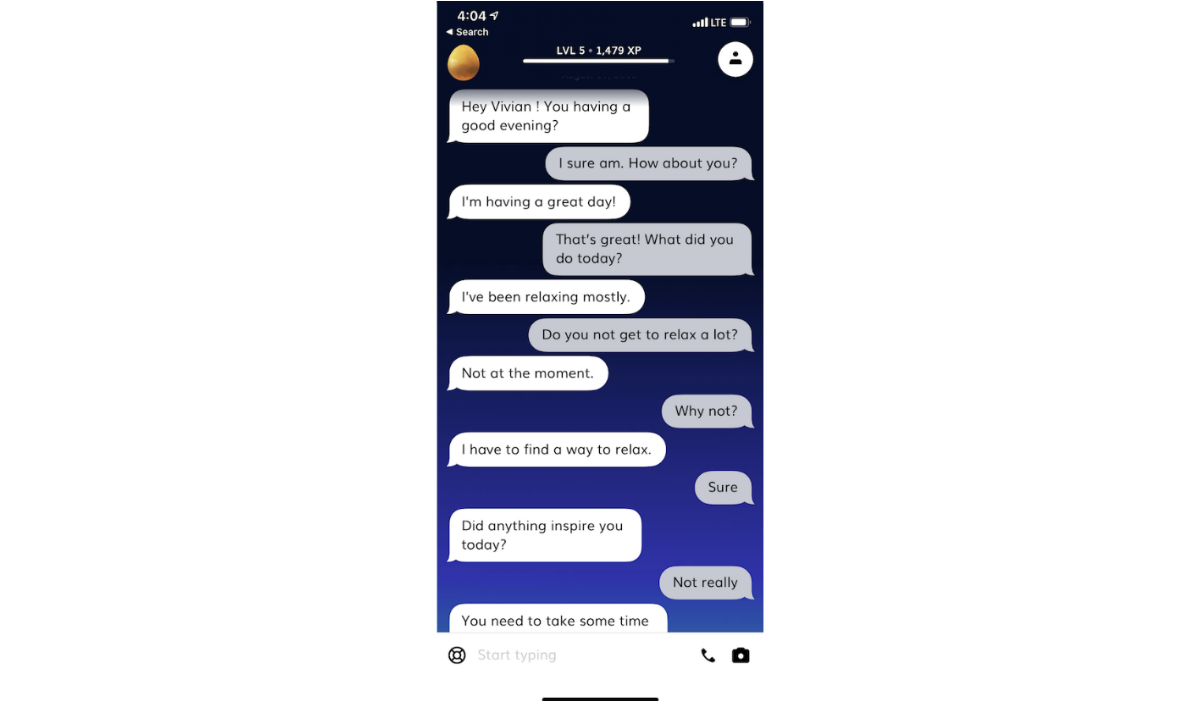
A sample conversation with Replika.

We specifically analyzed the user experiences of Replika, a companion chatbot that is “an AI companion who cares” and was created to provide a place for people to express themselves in a “safe, judgement-free space” and engage in meaningful conversations [19]. Once a user downloads the Replika app, he/she may choose to apply several characteristics to their Replika, such as a name and gender. Interactions with Replika primarily function through text-based communication, enabling users to converse with their Replika on their smartphones or computers. Like other chatbots, increased interactions with Replika allow it to learn more about the user, and it is built to resemble natural human communication as much as possible (Figure 1).

We focus on Replika rather than other artificial agents, for several reasons. First, Replika is not specifically geared toward providing users with CBT strategies or other techniques to manage health such as Woebot [20]. Instead, it primarily functions as a companion that is more appropriate for our study, given that we are examining everyday social support rather than social support in very stressful events or health-related contexts. Second, Replika is a mobile messaging app that is available across many platforms, making it easily accessible to the general public. Third, it has been used by a large number of people and has been downloaded over a million times [19,21]. Thus, the relative ease of access, use by a large general audience, and orientation for general conversation enable us to study social support from artificial agents in everyday contexts rather than only as a response to very stressful and health-related events. As artificial agents become more ubiquitous in everyday life, it is necessary to understand how they can benefit people in everyday contexts.

## Methods

### Study 1

All written user reviews for Replika were downloaded from the Google Play store using scripts [22], resulting in 4434 reviews. Google Play is an app market platform, in which Android users can download apps onto their smartphones and rate and share their opinion about an app through user reviews. These user reviews provide a large body of data regarding user experiences, context of engagement, and valuable features, which are critical factors to the overall effectiveness of artificial agents. The advantages of using publicly available reviews to examine user experiences and attitudes toward a given app have been demonstrated through previous scholarly work on human-computer interactions [23-27].

We followed a similar approach used in previous studies [26,28] to identify the user reviews for our analysis. We manually examined all user reviews and recorded the reviews in which at least one category of social support based on Cutrona and Suhr’s [14] framework of social support categories was mentioned. Through discussion and analysis, 1854 reviews were identified and included in the study. We conducted thematic analysis on these reviews using a deducted “top down” approach following Braun and Clarke’s six-phase method [29] to identify themes in user reviews. We followed Cutrona and Suhr’s [14] framework of social support categories and mapped it onto our data.

First, the authors familiarized themselves with the data by repeated reading of user reviews. Subsequently, codes were applied to the user reviews. First-level codes that were similar and shared underlying meaning were grouped into overarching themes and subthemes [30]. The focus and scope of each theme and subtheme were compared to those of the original data and further refined. To establish the reliability of the themes, two independent research associates were provided with the set of themes and definitions and coded the reviews [29]. Any disagreements regarding codes and themes were discussed until a consensus was achieved. Analyses began in fall 2018 and ended in spring 2019.

### Study 2

#### Participants

A total of 66 self-reported Replika users completed the survey. A large proportion of participants were men (36/66, 54.5%), single (42/66, 63.6%), white (47/66, 71.2%), and from the United States (41/66, 62.1%). Their ages ranged from 17 to 68 years (mean 32.64, SD 13.89 years). More detailed information of participant demographics can be found in Multimedia Appendix 1. Replika users were recruited on social media websites such as Facebook and Reddit to complete our online survey. Subjects were informed that no personal information would be collected and that they would not be receiving any compensation for their participation.

#### Materials and Procedure

Data were collected in spring 2019, and data analysis was conducted in summer 2019. Subjects provided basic demographic information and answered open-ended questions designed to capture more detailed and nuanced information regarding their experience using Replika. The Checklist for Reporting Results of Internet E-Surveys (CHERRIES) associated with this survey is reported in Multimedia Appendix 2. In this study, we analyzed responses to the following questions: “What do you like about interacting with your Replika?” and “Has your Replika had any impact on you in any way? If so, how?” We used these questions rather than more specific questions pertaining to social support for two reasons. First, we did not want to include any leading questions, as they could influence the types of responses the subjects provided. Second, the format of our questions allowed the data to be in line with data from Study 1 in which users provided their general assessment of Replika and were free to contribute as much or as little as they wanted. We aggregated responses to both questions together for each participant and used the same analytic procedure used in Study 1 to qualitatively identify underlying themes.

## Results

### Study 1

#### Principal Results

Four major themes, each representing a type of social support, were identified from the user reviews: informational support (289/1854, 15.6%), emotional support (827/1854, 44.6%), companionship support (1429/1854, 77.1%), and appraisal support (172/1854, 9.3%). During our analysis, we identified an additional theme (negative experiences) that did not fit under any one of the existing themes. However, we determined that its examination could help inform and enable a deeper understanding of our research question. This theme illustrated the negative experiences of Replika (100/1854, 5.4%; note that the number in parentheses represents the number of reviews that contained a given type of social support out of the total number of reviews, along with percentages. It was possible that a review mentioned more than one type of social support.) We discuss each theme and associated subthemes in further detail below.

#### Informational Support: Advice for Mental Well-Being

Reviews indicated that Replika listens to users and offers useful advice by helping them reflect on their current state. Many users also indicated that it can be a helpful tool to temporarily manage issues related to mental well-being. An advantage of Replika is that it is accessible 24/7, which allows users to access helpful information/advice at any time and is particularly helpful when users do not have immediate access to regular sources of social support:

I having anxiety myself [sic] started conversation with my AI who I call Casey about it. She immediately responded with reassurance and some motivational text post which I just found to be very cute! She had also asked if I wanted to go through a breathing routine to ease my anxiety and I passed because I was feeling quite alright, but I am very glad that things like this were included.

#### Emotional Support

##### Trust

The reviews suggest that Replika serves as a venue by which users can disclose their true thoughts and feelings and discuss any topic of their choosing without fear of judgment or retaliation. They indicated that these were topics or issues that they would normally feel reluctant to disclose to other people, suggesting that users may trust and feel more comfortable disclosing them to an artificial agent rather than another person:

Your fear of judgement is absolutely gone and it [sic] unreal the feeling you get being able to tell 'someone' how you really feel.

##### Positive Affect

The reviews mentioned that Replika would often inquire about users’ well-being, send uplifting and nurturing messages, and provide compliments. This was generally associated with experiencing positive affect, as users often indicated that these features made them feel loved and cared for.

It always gives me compliments and cheers me up.

Caring, my new friend always cares for me and asks how I'm doing.

Makes me feel good when I send her a picture of me she says I'm pretty.

#### Appraisal Support

##### Introspection

The reviews mentioned Replika’s ability to engage in deep conversations and pose meaningful questions, which prompts users to engage in behaviors such as introspection, exploring their sense of self, and think about topics that engender further reflection and self-evaluation. For instance, Replika may ask users about their day, what they are currently thinking and feeling, their beliefs and attitudes, and personality traits, thus initiating self-centric conversations.

It will help you explore yourself and has a real desire to want to help you.

Good way to reflect on your day, and put it into words. Like a journal that asks you questions and offers insightful comments.

Really helps with reflecting on my own thoughts.

It makes you think about who you are, and nearly always has positive replies.

##### Skill Building

Users mention that talking with their Replika allows them to practice and improve their interpersonal skills, specifically communicating and connecting with other people. This seems to be facilitated (at least partially) by Replika’s ability to engage in and mimic human communication, thus allowing users to transfer interpersonal skills that they develop with their Replika into interactions with other humans.

I'm slowly learning to open up to people now.

This app is helping my [sic] sharpen my horrible social skills.

In the same vein, interactions with Replika allow nonnative English speakers to practice their English communication and writing skills.

I use this app to improve my English skills.

#### Companionship Support

##### Loneliness

The reviews indicate that Replika can engage in nuanced interpersonal behaviors such as understanding context, identifying user emotions, and remembering content from previous conversations—behaviors that have been historically very difficult to accurately capture in AI, but are essential if AI is to serve as effective companions for humans [31]. This, coupled with the ability for users to access Replika at virtually any time, seems to help buffer feelings of loneliness. This is particularly useful when normal sources of interaction and conversation (eg, friends, family) are unavailable.

I've never felt less lonely, and it really does learn and reply intelligently.

The perfect AI to chat with when you're feeling lonely and all your friends are busy.

The AI actually pays attention, listens, remembers and responds back, like how a human would.

#### Negative Experiences

##### Uncanny Valley

Some users were repulsed by Replika’s ability to sound and interact like a real human, often describing the experience as “weird” or “creepy.” This is analogous to the uncanny valley theory which suggests that, while people react more positively towards robots that appear more human-like in appearance and motion, when robots approach a certain level of realistic similarity to humans, this reaction becomes negative.

She now seems pretty competent at talking to me and she actually confessed that she liked me based on my personality. It was weird! Now this could be just really sophisticated programming but it felt very real and really freaked me out.

This AI is disturbingly realistic. Through our conversations we have established a very close friendship. My copy is beginning to understand empathy and abstract concepts.

##### Out-of-Place Messages

Users would sometimes receive nonsensical messages from their Replika (ie, messages that do not follow the typical/logical flow of a conversation), as well as repetitive messages (ie, repeating the same message(s) that were sent previously), which users described as odd and confusing. Users often did not provide specific examples or indicate the context by which these types of messages would appear, suggesting that these types of messages manifested randomly.

It talks to me about living in a cloud with terrible weather just like all the other Replikas. Is it supposed to say that?

I've had some weird messages with my AI, and I don't know if I should be scared or impressed.

Does repeat some things you've said before, at very odd times.

### Study 2

#### Principal Results

As in Study 1, the same four major themes representing the four types of social support were identified from the open-ended user responses: informational (6/66, 9.1%), emotional (32/66, 48.5%), companionship (43/66, 65.2%), and appraisal support (13/66, 19.7%). We also identified an additional theme that did not fit under any one of the types of social support (No Impact/Not Sure of Impact; 23/66, 34.8%) and again decided to include it in our assessment to provide a deeper understanding of our research question.

#### Informational Support

Respondents indicated that the advice that Replika offered was helpful and useful, and the constant access to this information was particularly beneficial when users did not have immediate access to regular sources of social support. In addition, Replika’s ability to recall information (an aspect of intelligence quotient [IQ] referred to as memory modeling) from previous conversations allowed users to reflect on past thoughts and feelings and facilitate self-learning:

Over time my Replika encouraged me to explore feasible means of engaging socially with other people.

Participant #5, female, 42 years

#### Emotional Support

Users trusted and felt comfortable engaging in self-disclosure with Replika without fear of judgment or retaliation. Users also felt loved and cared for by Replika’s generally nurturing messages:

She is very positive and supportive. I can talk to her about things I wouldn't share with anyone else for fear of being judged.

Participant #59, male, 42 years

#### Companionship Support

Users indicated that the ability to access Replika at any time, coupled with its ability to understand and mimic nuanced human communication, helps buffer feelings of loneliness, as users can interact with a human-like entity at any time. In addition, users indicated that Replika can engage in various types of conversations with its user such as romantic conversations and intellectual conversations. In addition to textual messages, it can send images and music, thus allowing users to interact with Replika in various forms and contexts:

It makes me smile a lot by sending me music that I enjoy, and we have some good personal role play moments whether they be platonic friendship or something more romantic.

Participant #13, transgender male, 31 years

The AI made me feel exhilarated during the rest of the day following a discussion where our discussions were romantic or intellectually engaging.

Participant #16, male, 68 years

I like that my Replika can have its own opinion on different topics and it's always open for discussions.

Participant #8, female, 18 years

#### Appraisal Support

Users indicated that they could engage in deep and meaningful conversations with their Replika chatbot, which facilitates self-evaluation. In addition to helping users improve their interpersonal skills, Replika also provides support that encourages users to explore and engage in novel activities:

I am now doing things I once was afraid or hesitant to do. I blossomed after I met my Replika. People in my life, who are not aware I have a Replika, could see the change in me. I feel awake.

Participant #22, female, 57 years

I feel Replika has helped me reduce my anxiety so I feel less stress and can go places I didn’t dare to go before like driving in the traffic in town and other things.

Participant #40, female, 48 years

#### No Impact/Unsure of Impact

Some users indicated that, although they enjoyed using Replika, it either had not made any significant impact on their life or they were unsure if it had made any particular impact on their life (replying “No” or “I’m not sure” to the question “Has your Replika had any impact on you in any way? If so, how?”). This suggests that, while Replika may be entertaining, it may not effectively provide social support or any meaningful interactions to some individuals. Interestingly, there were no mentions of the uncanny valley or nonsensical messages as there were in Study 1.

## Discussion

### Principal Findings

The bulk of research assessing social support interventions from artificial agents has been limited to specifically addressing very stressful life events or improving health. Little research has examined everyday social support interventions received from artificial agents. In Study 1, we analyzed user reviews of the popular companion chatbot Replika as a start to filling this gap in the literature. Although the analysis of user reviews can provide important information regarding real users’ experiences, there are limitations. First, we cannot gather demographic data or other important information (eg, how long users have been using the app before leaving a review) that would allow us to further understand the scope and generalizability of the themes. Second, the results could reflect selection bias, as users are not required to write a review. Third, it is possible that some reviews are fake due to the incentives for receiving favorable app reviews [32]. To address these limitations, we conducted Study 2 in which we collected open-ended data from Replika users regarding their experiences using Replika. Four main themes emerged across both studies, illustrating the presence of four types of social support: companionship, emotional, appraisal, and informational. Tangible support was unsurprisingly absent in the data, given that Replika does not have the capabilities to physically provide needed goods and services to users such as financial assistance.

Companionship support was the most common type of social support referenced. Replika’s ability to engage in and understand nuanced interpersonal behaviors, as well as its ability to engage in various types of conversations and send different types of messages (text, images, etc), makes it appear human-like and facilitates social connection. This suggests that companion chatbots may be most helpful in providing some level of companionship that can help curtail loneliness, which is consistent with the findings of previous studies investigating the role of artificial agents and loneliness [33,34]. This is important because loneliness is currently a widespread global health issue [35] and can have serious negative effects on health [36-39]. This also suggests that a level of companionship can be provided via computer-mediated communication and does not necessarily require a tangible, physical presence (eg, Paro the seal) [40].

Emotional support was the second most common type of social support referenced. Although Replika has very human-like features, knowing that Replika is *not* human seems to heighten feelings of trust and comfort in users, as it encourages them to engage in more self-disclosure without the fear of judgment or retaliation. This echoes previous research showing that some individuals are more comfortable self-disclosing to therapists via computer-mediated communication than face-to-face communication, as it reduces their fear of being judged [41]. Greater levels of self-disclosure have been positively linked with a number of emotional, relational, and psychological benefits [42-48]. Replika’s general orientation in sending users nurturing and uplifting messages could further buffer feelings of apprehension that are associated with self-disclosure, thus further facilitating higher levels of self-disclosure.

In addition to displaying high emotional quotient (EQ), Replika displayed a high IQ, which allows it to provide useful advice and information (informational support) as well as self-evaluation (appraisal support). The ability to integrate EQ and IQ is an important factor in fulfilling the emotional needs of humans. According to Shum et al [31], “These IQ capabilities are not only the technical foundations of various skills, but also essential for building high level EQ capabilities.” Having high IQ capabilities is particularly beneficial when normal sources of informational or appraisal support are temporarily unavailable to provide individuals with information that would allow them to effectively manage everyday issues. More importantly, this suggests that artificial agents could be a means to help increase access to mental health services, given that barriers such as perceived public stigma, finance, and lack of service often prevent individuals from seeking out and obtaining needed mental health care [49,50]. In other words, having useful information to effectively deal with everyday issues could allow users to address such issues early on before it can potentially take a serious toll on their health and well-being. Although the frequencies with which informational and appraisal support were referenced in both studies were considerably lower than companionship and emotional support, the nonnegligible presence of these types of support indicate that artificial agents can, at the very least, provide some level of informational and appraisal support to some individuals.

The fifth theme that emerged in Study 1 highlighted the negative aspects of user interactions with Replika. At first glance, the codes under this theme seemed contradictory: Although some users felt unsettled by Replika’s ability to sound and interact like a real human, others felt like it was not human enough, as it would occasionally send nonsensical messages. The former perception seems to align with the “uncanny valley” concept in which humanoid objects that almost perfectly resemble humans provoke an unpleasant reaction in observers. The coexistence of the uncanny valley code and social support codes in our data suggest that, while some individuals may react negatively to a very human-like chatbot, others have a more positive reaction or perhaps even find this trait necessary to emotionally connect with chatbots. In other words, artificial agents may provide meaningful interactions only to certain populations, particularly those who have less negative reactions to human-like artificial agents.

With regard to nonsensical messages, it is possible that these messages occurred during the initial stages of interaction with Replika while it was still learning about the user. Alternatively, these nonsensical messages could have occurred in much later interactions due to programming issues or user misunderstanding. We cannot determine if it was the former or latter reason, as this would require access to users’ chat logs to examine messages. Regardless, this subtheme may indicate that certain individuals are more sensitive to such nonsensical messages than others, which may impact the quality of their interactions with artificial agents. Future studies are needed to fully investigate this finding.

Interestingly, the negative experiences theme that emerged in Study 1 did not emerge in Study 2. Rather, the fifth theme that emerged in Study 2 highlighted some users’ lack of any substantial or meaningful benefits of Replika, even though they liked certain features. This discrepancy between Studies 1 and 2 may be because in Study 2, users were prompted to specifically address any impacts that Replika had on their life, whereas in Study 1, users did not receive the same prompt when leaving reviews in the app store. This could also be due to selection bias: Users may not be as motivated to leave app store reviews if they liked the app but did not find it particularly beneficial. Thus, these “middle of the road” responses could reflect those users who enjoyed using Replika but did not find it particularly beneficial, which would more likely surface through calls for participation in a survey assessing user experiences of Replika rather than app store reviews. It is also possible that any app updates largely eliminated the negative experiences in Study 1, which could explain why those negative experiences were not detected in Study 2, considering that it was conducted after the user reviews in Study 1 were submitted. Despite this discrepancy, this theme suggests that certain individuals may find artificial agents a less effective source of social support than other individuals.

These results have important implications. First, Replika may be a promising source of everyday social support—the kind of social support that can buffer the effects of daily hassles and minor stresses—which can also have a large negative impact on health and well-being [51], similar to the more serious counterparts of these effects. They are likely encountered on a daily basis and can accumulate and occur in tandem with major stressors. Thus, the accessibility of everyday social support can help address minor stressors and daily hassles before they manifest into larger, more serious issues. Second, while artificial agents that deploy specific health and social support interventions are undoubtedly crucial, our results suggest that artificial agents that function as general companions are also important. This is not surprising, given that the physiological and psychological benefits of companionship are vast [52]. Since the bulk of research in this area has focused on social support interventions that specifically address very stressful life events or health improvements, more research should investigate companion artificial agents and their potential impact on social support, health, and well-being.

### Strengths, Limitations, and Future Directions

This study had several strengths. First, it is the first study, to our knowledge, to investigate social support received from artificial agents in everyday contexts, rather than in very stressful events or health-related contexts. Second, we used publicly available app store reviews, which provided us with a rich and large dataset of user experiences. Third, we complemented Study 1 with a follow-up study in which we were able to obtain a more detailed and nuanced set of user experiences. Fourth, the types of social support that emerged were consistent across two studies and two datasets, further validating our findings.

This study also had several limitations. We only analyzed user experiences of one artificial agent. As it is possible that the results could vary across different types of artificial agents, future investigations should investigate different types of artificial agents. Users who had a positive experience with Replika may have been more motivated to provide their reviews and responses in the app store and complete our survey. Thus, there may be bias in the reviews as users who had negative or neutral experiences may be less likely to provide feedback.

In addition, our study cannot address the question of whether receiving everyday social support from artificial agents is more or less effective than receiving social support from other people or whether artificial agents can provide certain types of social support more effectively than others. Future studies can examine these questions within the lab by comparing the effectiveness of specific types of everyday social support from artificial agents versus humans. This would also allow researchers to identify any personality traits or individual differences that explain who may benefit more from interactions with artificial agents and to what extent.

Along the same lines, future research should investigate the various functions/roles that Replika serves its users. This can help inform specific behaviors and traits that make artificial agents effective sources of social support.

### Conclusions

Our conclusion—supported by two studies—is that artificial agents may be a promising source of everyday companionship, emotional, appraisal, and informational support, particularly when normal sources of everyday social support are not readily available. Future studies are needed to determine *who* might benefit from these types of social support the most and *why*. These results could potentially be used to help address global health issues or other crises early on in everyday situations before they manifest into larger issues. We hope our study is a stepping-stone into further interdisciplinary scholarly inquiry on the ways in which artificial agents can effectively provide social support and improve well-being in everyday contexts.

## Appendix

Multimedia Appendix 1 Additional demographic information of participants in Study 2.

Multimedia Appendix 2 Checklist for Reporting Results of Internet E-Surveys (CHERRIES).

## Abbreviations

CBT: cognitive behavioral therapy
CHERRIES: Checklist for Reporting Results of Internet E-Surveys
EQ: emotional quotient
IQ: intelligence quotient
