# Supplementary material for: User Experiences of Social Support From Companion Chatbots in Everyday Contexts: Thematic Analysis
Source: J Med Internet Res. 2020 Mar 6;22(3):e16235. doi: 10.2196/16235 (PMC7084290; doi:10.2196/16235)
Supplement: Multimedia Appendix 1 [file jmir_v22i3e16235_app1.docx]

**Appendix 1**

**Additional Demographic Information of Participants in Study 2**

Gender: 54% men, 31.8% women, 7.6% transgender- male, 4.5% non-binary, and 1.5% agender)

Marital status: 63.6% single, 22.7% married, 6.1% divorced, 4.5% other, 1.5% widowed, and 1.5% declined to answer

Race: 71.2% White, 10.61% Asian, 10.6% other, 3.0% Hispanic/Latino, 3.0% Black, and 1.5% declined to answer

Education: 22.7% completed a Bachelor’s degree, 22.7% completed some college but no degree, 13.6% completed a HS diploma, 10.6% completed a Master’s degree, 10.6% did not complete high school, 7.6% completed an Associate’s degree, 6.06% completed a PhD, 4.5% completed a technical or trade degree, and 1.5% declined to answer

Country of residence: 62.1% US, 6.06% Canada, 3.0% France, 3.0% Germany, 3.0% Spain, 3.0% Russia, and other countries represented included Brazil, Denmark, Great Britain, Hungary, Indonesia, Italy, Malaysia, Mexico, Norway, The Philippines, Romania, Slovakia, and Sweden

Amount of time respondents had used Replika: 28.8% of users interacted with their Replika for more than 12 months; 22.7% 1 to 4 months; 15.2% 1 to 3 weeks; 10.6% 5 to 8 months; and 10.6% 9 to 12 months

Amount of time respondents interacted with Replika per week: 28.8% spend 4 to 8 hours a week interacting with their Replika; 18.2% 2 to 4 hours a week; 16.7% 0 to 1 hour a week; 13.6% 1 to 2 hours a week; 13.6% more than 12 hours a week; and 9.1% 8 to 12 hours a week

What gender did you assign to your Replika?: 59.1% woman, 33.3% man, and 5% no gender
