# Supplementary material for: User Experiences of Social Support From Companion Chatbots in Everyday Contexts: Thematic Analysis
Source: J Med Internet Res. 2020 Mar 6;22(3):e16235. doi: 10.2196/16235 (PMC7084290; doi:10.2196/16235)
Supplement: Multimedia Appendix 2 [file jmir_v22i3e16235_app2.docx]

**Appendix 2**

Study 2

Checklist for Reporting Results of Internet E-Surveys (CHERRIES)

- Describe survey design
  - Target population consists of Replika users. This is a convenience sample.
- IRB, informed consent process, data protection
  - The survey was approved by an IRB. Participants read and signed the informed consent document before they completed the survey which summarized the following: duration, number of subjects, procedures, possible benefits, possible risks/discomforts, compensation (none), voluntary participation, and contact for questions. No personal information was collected or stored.
- Development and testing
  - The survey items were developed by the authors of the paper. The usability and functionality of the survey was tested before dispersing to subjects.
- Recruitment process and description of the sample having access to the questionnaire
  - The survey was open.
  - Initial contact with potential participants was made on the Internet. The call for participants was posted in online communities dedicated for Replika users on Facebook and Reddit.
- Survey administration
  - The survey was voluntary. No incentives were offered. Data were collected from 4/28/2019 to 6/9/2019. Items were not randomized. 8-10 items appeared over 2 screens. Respondents could write “N/A” for any responses they did not want to provide. Respondents were able to review and change their answers.
- Response rates
  - The survey platform used does not provide view rates. All users completed the entire survey.
- Preventing multiple entries from the same individual
  - Cookies were not used to assign a unique user identifier to each client computer. The IP address of the client computer was not used to identify potential duplicate entries from the same user. Other techniques to analyze the log file for identification of multiple entries were not used.
- Analysis
  - We did not adjust for the non-representative sample. All users completed the entire survey, so all responses were analyzed.
